# The meta-analysis of the effect of ^68^Ga-PSMA-PET/CT diagnosis of prostatic cancer compared with bone scan

**DOI:** 10.1097/MD.0000000000025417

**Published:** 2021-04-16

**Authors:** Ruining Zhao, Yajie Li, Lihong Nie, Kaiyue Qin, Hang Zhang, Hongbin Shi

**Affiliations:** a Department of Urology, General Hospital of Ningxia Medical University; b Department of Physiology, School of Basic Medical Sciences, Ningxia Medical University, Yinchuan, Ningxia, PR China.

**Keywords:** ^68^Ga-PSMA-positron emission tomography/computed tomography, bone metastasis, bone scan, prostatic cancer, meta-analysis

## Abstract

**Background:**

^68^Ga-PSMA-PET/CT (positron emission tomography/computed tomography) is a promising method for prostate cancer (PC) detection. However, the ability of ^68^Ga-PSMA-PET/CT to detect malignant bone lesions, and whether this method is superior to the existing bone imaging methods are still lack of systematic analysis.

**Purpose:**

To evaluate the value of ^68^Ga-PSMA-PET/CT and bone scan in clinical diagnosis of prostatic cancer from the perspective of evidence-based medicine.

**Methods:**

PubMed, The Cochrane Library, EMBASE, Springer Link, Sinomed, CNKI, Wanfang database, and CQVIP database were searched to find the satisfactory studies that needed systematic review of trials and compared the value of 68Ga-PSMA-PET/CT and bone scan. All studies published from inception to March 31, 2020. According to the inclusion and exclusion criteria, 2 reviewers independently evaluated and extracted the literature. Review Manager 5.3 was applied to evaluate the included literature quality. The heterogeneity of the included literature was tested by Meta Disc 1.4, and the effect model was selected according to the heterogeneity test results, and the sensitivity (SEN), specificity (SPE), PLR, NLR and diagnostic odds ratio (DOR) were analyzed. After testing the heterogeneity results of literature by using the 95% confidence interval and the forest map.

**Results:**

A total of 4 studies were eligible for inclusion in the meta-analysis, which included 318 patients, 120 cases with bone metastasis and 198 cases without bone metastasis. The results of summary evaluation for ^68^Ga-PSMA-PET/CT and bone scan in diagnosis of prostatic cancer as follow respectively: The SEN were 0.97 and 0.86; the SPE were 1.00 and 0.87; the DOR were 1468.33 and 36.23; PLR were 88.45 and 6.67; NLR were 0.05 and 0.19; and the area under curve (AUC) and 95% CI were 0.9973 (1.0000–0.9927) and 0.8838 (0.9584–0.8092).

**Conclusion:**

By comparing the diagnostic results of ^68^Ga-PSMA-PET/CT and bone scan imaging diagnosis methods, the ^68^Ga-PSMA-PET/CT has a higher SEN and SPE than bone scan, and it has a higher diagnostic efficiency for prostate cancer bone metastasis, which is worthy of clinical application.

## 1 Introduction

Prostate cancer (PC) is one of the most common malignancies in men worldwide, according to the American Cancer Society report, PC ranks first in the annual incidence rate, the death rate is only next to lung cancer. With the increase of age, the incidence rate of PC is still increasing.^[1]^ PC are particularly prone to osseous involvement, which occurs in about 30% of cases.^[2]^ Bone metastasis can lead to osteoblastic and/or osteoclastic focuses, and PC is predominantly osteoblastic focuses in nature. PC cells secrete transforming growth factorβ, insulin-like growth factor, platelet-derived growth factor, endothelin 1, urokinase plasminogen activator, etc, which regulate osteoblast proliferation and differentiation. Osteoblasts secrete receptor activator of the nuclear factor-κB ligand (RANKL) and interleukin 6 to activate osteoclasts, which elicits bone resorption and secrete epidermal growth factor and calcium, thereby stimulating cancer cell proliferation in bone. It formed a vicious circle in the bone microenvironment of bone metastatic PC. The degree of metastatic bone involvement has been shown to be an independent prognostic factor in PC patients,^[3]^ Bone metastasis will seriously affect the quality of life of patients. Early diagnosis of bone metastasis and symptomatic treatment will effectively reduce the probability of bone related adverse events and improve the quality of life of patients.^[4]^ X-ray, computed tomography(CT), magnetic resonance imaging(MRI), ^99^Tc^m^ methylene diphosphonate whole body bone scintigraphy, positron emission tomography/computed tomography (PET/CT) and single photon emission computed tomography/computed tomography(SPECT/CT) are commonly used in the diagnosis of bone metastases, each of which has its own advantages and disadvantages. Bone scan is a sensitive and relatively cheap method to detect bone metastasis in prostate cancer patients, but its specificity (SPE) is limited. Many benign bone diseases can simulate bone metastasis in bone scan, and this examination has insufficient sensitivity (SEN) to early pathological changes involving bone marrow and osteogenic activity. PSMA also known as folate hydrolase I or glutamate carboxypeptidase II, is a type II, 750 amino acid transmembrane protein, which is highly overexpressed (100–1000 fold) on almost all PC tumors. Small molecule PSMA inhibitors bind to the active site in the extracellular domain of PSMA and are internalized and endosomally recycled, leading to enhanced tumor uptake and retention and high image quality.^[5]^ A variety of inhibitors have been synthesized, but ^68^Ga labeled radiotracers are the most widely used because of their high tumor contrast,^[6]^ good diagnostic performance in the detection of lymph nodes, distant metastasis and primary tumors.^[7,8]^ In recent years, there are more and more comparisons between ^68^Ga-PSMA-PET/CT and bone scan in the diagnosis of bone metastasis of prostate cancer, but there is still a lack of systematic analysis. This study integrated the relevant literature from inception to March 31, 2020, and carried out a systematic analysis, in order to provide high-level evidence for clinical diagnosis and treatment decisions.

## 2 Materials and methods

### 2.1 Retrieval strategy

A systematic search of PubMed, The Cochrane Library, EMBASE, Springer Link, Sinomed, CNKI, Wanfang database, and CQVIP was performed to identify all studies published from inception to March 31, 2020. The searches included the search words “prostate cancer OR prostate carcinoma” and “bony metastases OR skeletal metastases OR osseous metastases” and “bone scan OR bone scintigraphy” and “PET/CT OR positron emission tomography/CT” and “psma OR prostate specific membrane antigen” in the all text. There were no restrictions with respect to the language, date of coverage, study types, methodology, or geographical limits. The “related articles” function and the reference lists of included articles were used to broaden the search.

### 2.2 Inclusion and exclusion criteria

#### 2.2.1 Inclusion criteria

The studies must include the following requirements:

1. randomized controlled trail and retrospective cohort study;
2. The patients were diagnosed as PC (with or without bone metastasis);
3. All patients received ^68^Ga-PSMA-PET/CT and bone scan, the gold standard is determined by pathological analysis and/or clinical and imaging follow-up;
4. study compared the diagnostic value of ^68^Ga-PSMA-PET/CT and bone scan.
5. studies provided original diagnostic data (SEN, SPE), positive likelihood ratio [+LR], negative likelihood ratio [–LR], Diagnostic odds ratio [DOR], area under curve [AUC]) or can be calculated using enough evidence;
6. The report time of the study is from inception to March 31, 2020.

#### 2.2.2 Exclusion criteria

Exclude the study if the following exclusion criteria are met:

1. The inclusion criteria were not met;
2. Incomplete or unable to extract research data (study on the number of true positive, false positive, false negative, and true negative cases that can not be calculated completely);
3. Case report, reviews, case analysis, meeting abstract, no full-text studies, and comment literature;
4. ^68^Ga-PSMA-PET/CT and bone scan were not performed at the same patients.

### 2.3 Literature quality evaluation and data extraction

Two reviewers independently evaluated the study's eligibility based on inclusion/exclusion criteria. If there are differences, they will consult for resolution or a third reviewers will provide help. The standard procedure was performed to extract data from the studies. Two reviewers independently extracted the following participant characteristics: The first author, publication time, study interval, research design, average age of patients, numbers of cases, (SEN) and SPE data, true positives, false positives, true negatives, false negatives, diagnosis standard. The methodological quality of the studies included in our meta-analysis was assessed using the quality assessment of diagnostic accuracy studies (QUADAS-2) checklist by RevMan software version 5.3 for Windows. Disagreements were resolved by discussion or consensus with a third member of the team.

### 2.4 Statistical analysis

Data were initially entered and analyzed using Meta Disc software version 1.4 for Windows.

The following measures of test accuracy were computed to assess the accuracy of ^68^Ga-PSMA-PET/CT and bone scan for the diagnosis of bone metastasis: SEN, SPE, +LR, -LR, and DOR. The SROC curves were pooled to evaluate the overall diagnostic performance. Chi Squared test was used for SEN and SPE heterogeneity test, and Cochran Q test was used for DOR, +LR, -LR. The heterogeneity of each index was divided into 3 categories. *I*^2^ < 50% indicates low heterogeneity, 75% > *I*^2^ ≥ 50% indicates medium heterogeneity, and *I*^2^ ≥ 75% indicates high heterogeneity. When *I*^2^ < 50%, evidence shows no significant heterogeneity, use fixed-effects model, On the contrary, the random effects (RE) model is adopted.^[9]^

## 3 Results

### 3.1 Search results and quality assessment

**Figure 1:**
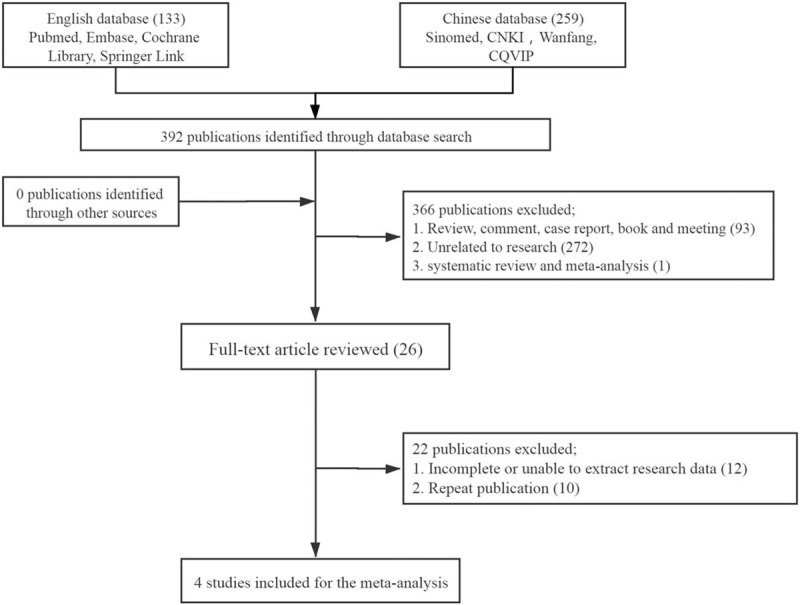
Flowchart of study inclusion.

A total of 392 documents were retrieved from the database. According to the eligibility criteria, 366 ineligible records were excluded by screening titles and abstracts. Subsequently, Full-text of all potentially relevant trials were downloaded for full-text review. After detailed search and selection, 4 papers were finally obtained, which included 3 English literature, 1 Chinese literature, 318 patients in total, 120 in bone metastasis group and 198 in nonbone metastasis group. Figure 1 shows the article selection process. Two reviewers independently extracted the following participant characteristics: The first author, publication time, study interval, research design, average age of patients, numbers of cases, SEN, SPE, +LR, -LR, and DOR. The main characteristics of the 4 studies are shown in Table 1.^[10–13]^

**Table 1:** The main characteristics of the 4 studies.

					^68^Ga-PSMA-PET/CT				Bone scan				Diagnosis standard
First author	Year	Study type	Age mean	No. of patient	TP	FP	FN	TN	TP	FP	FN	TN	
Kumar	2018	PRO	-	74	49	0	2	53	47	1	4	22	PET/CT, Bone scan, Pathology, Clinical and imaging follow-up
Lengana	2018	PRO	66	113	25	0	1	87	19	11	7	76	PET/CT, Bone scan, CT, MRI, Pathology, Clinical and imaging follow-up
Song	2018	RE	69.1	73	32	0	0	41	29	2	3	39	PET/CT, Bone scan, CT, MRI, PSA, Clinical and imaging follow-up
Uslu-Besli	2019	RE	67.3	28	10	0	1	17	8	8	3	9	PET/CT, Bone scan, CT, MRI, Clinical and imaging follow-up

**Figure 2:**
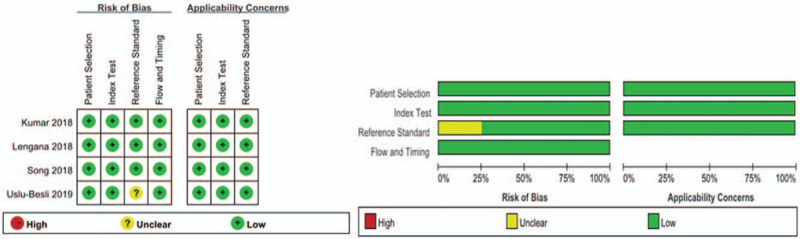
The methodological quality graph.

The methodologic and reporting quality of the studies by use the QUADAS-2 checklist of RevMan software version 5.3 for Windows. The methodological quality graph is shown in Figure 2. Generally speaking, the quality of the literature included in the study is better, the risk of bias is low, and the applicability is good.

### 3.2 Heterogeneity test

The results of heterogeneity test showed that the (SEN) (*I*^*2*^ = 0.7%), SPE (*I*^*2*^ = 0.0%), DOR (*I*^*2*^ = 0.0%), PLR (*I*^*2*^ = 0.0%), and NLR (*I*^*2*^ = 0.0%) of ^68^Ga-PSMA-PET/CT had low *I*^*2*^ value, which was suitable for fixed effect model. The (SEN) (*I*^*2*^ = 55.8%), SPE (*I*^*2*^ = 82.0%), DOR (*I*^*2*^ = 80.6), PLR (*I*^*2*^ = 88.6%), and NLR (*I*^*2*^ = 70.1%) of bone scan had high *I*^*2*^ value, which were suitable for the random effect model. The results show that the heterogeneity is caused by the random error due to the different research methods included in the study, the different occurrence time of each study, the individual and regional differences of research objects, and the different diagnostic threshold adopted by different studies.

### 3.3 publication bias

Owing to the limited number (below 10) of studies included in each analysis, publication bias was not assessed.

### 3.4 Outcomes of meta-analysis

**Figure 3:**
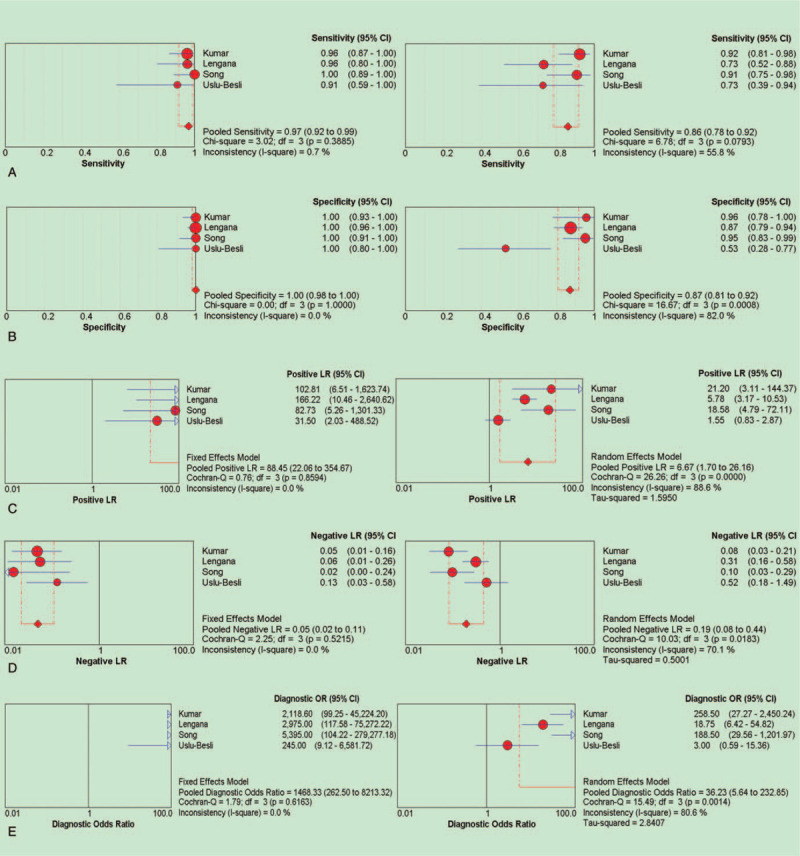
Forest plots of the pooled SEN (A), SPE (B), +LR (C), -LR (D), and DOR (E) of ^68^Ga-PSMA-PET/CT and bone scan for the diagnosis of bone metastasis.

**Figure 4:**
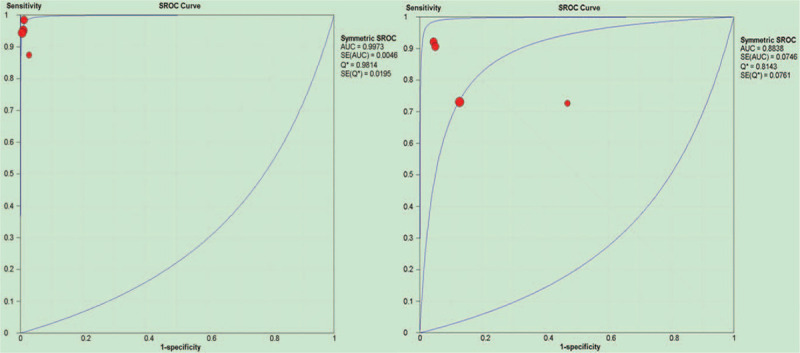
Forest plots of the SROC of ^68^Ga-PSMA-PET/CT and bone scan for the diagnosis of bone metastasis.

The pooled SEN and SPE were 0.97 and 1.00 vs 0.86 and 0.87 for ^68^Ga-PSMA-PET/CT and bone scan. According to the analysis results, the (SEN) and SPE of ^68^Ga-PSMA-PET/CT in the diagnosis of bone metastasis were superior to that of bone scan, the +LR for ^68^Ga-PSMA-PET/CT and bone scan were 88.45 and 6.67, -LR were 0.05 and 0.19, DOR were 1468.33 and 36.23, AUC and 95% CI were 0.9973(1.0000–0.9927), and 0.8838 (0.9584–0.8092), respectively (Figs. 3 and 4). Together, these data demonstrate that the diagnostic value of ^68^Ga-PSMA-PET/CT is of a stroke above that of bone scan.

## 4 Discussion

With the development of PC, most patients will have bone metastasis. The complications of bone pain, pathological fracture, and spinal cord compression caused by bone metastasis will seriously affect the quality of life and prognosis of patients. Therefore, early and accurate diagnosis is necessary to determine the most effective treatment plan for patients, and may increase the expected 5-year survival rate to 100%.^[14]^ The gold standard for the diagnosis of bone metastases is pathological diagnosis, which requires biopsy of every suspicious focus, which has great damage to patients and is difficult to obtain the consent of patients. Therefore, comprehensive application of imaging examination is the main trend in the diagnosis of osseous metastases. Cross-sectional functional images are produced by SPECT or PET, while cross-sectional anatomical images can be obtained with either X-ray, CT, or MRI. Both X-ray and CT can reflect changes in bone structure and density. X-ray has the advantages of economy, easy operation, wide examination range, and high spatial resolution. It is of high diagnostic value for lesion size, periosteum reaction, cortical bone continuity and morphological changes. However, X-ray sensitivity and SPE were low, and the false negative rate for bone metastases was up to 50%.^[15]^ The scope of CT examination is limited, but the fine structure of the lesion can be found, and the (SEN) is higher than that of X-ray. Bone scans usually diagnose bone metastases 3 to 6 months earlier than X-ray and CT. MRI, especially WB-MRI, has been widely used in the detection, staging, follow-up and evaluation of response to treatment of prostate cancer. It can provide both anatomical images and functional images while avoiding radiation damage. Padhani et al found that WB-MRI is significantly better than bone scan in detecting bone metastases of prostate cancer.^[16]^ In 2016, the European Association of Urology (EAU) put forward a clear specification for the application of WB-MRI in prostate cancer.^[17]^ Bone scan can present the whole body image at 1 time, reflect the information of bone blood supply and bone metabolism, and has the widely available and cost-effective procedure of bone scintigraphy. So, bone scan has been performed to evaluate bone metastasis for staging, follow-up, and treatment response assessment.^[18]^ If the bone metastases of prostate cancer are small and few, the bone scan will show a highly concentrated focus, which is easily affected by benign bone lesions, such as fractures and bone degeneration.^[19]^ SPECT/CT uses CT to scan the region of interest on SPECT, with high resolution and high (SEN). Compared with SPECT, SPECT/CT has the advantages of accurate positioning and high resolution in the diagnosis of bone metastases. It can identify the location and size of the lesion, show the anatomical relationship between the lesion and the surrounding tissues, and clarify the tumor invasion area.^[20]^ Horger et al found that the SEN and SPE were 0.98 and 0.81 vs 0.94 and 0.19 for SPECT/CT and SPECT.^[21]^ SPECT/CT has a SPE significantly improved. PET has high SPE and (SEN), but its spatial resolution is poor. The fusion of PET and CT can provide not only anatomical information, but also functional information, thereby improving diagnostic efficiency. PET/CT can not only detect bone metastases, but also image whole body organs. Qu et al believe that the SEN and SPE of PET/CT are better than MRI and bone scan.^[22]^ PSMA is overexpressed in most prostatic adenocarcinomas, and its expression levels increase with increasing tumor dedifferentiation in hormone-refractory and metastatic PC.^[23,24]^ At present, small molecule inhibitors often used as the molecular probes of PSMA. ^68^Ga is used to label small molecule inhibitors, which are distributed throughout the body with the blood and concentrated in prostate cancer tissues with high expression of PSMA, so we can get the images of the primary and metastasis of prostate cancer. With the development of medical imaging technology, ^68^Ga-PSMA-PET/CT was successfully used in prostate cancer patients in 2012,^[25]^ and showed good application value in the diagnosis, staging and recurrence of prostate cancer.^[26]^ At present, there are more and more comparisons between ^68^Ga-PSMA-PET/CT and bone scan in the diagnosis of bone metastasis of prostate cancer, but there is still a lack of systematic analysis. This study integrated the relevant literature of each database from inception to March 31, 2020, and carried out a systematic analysis. The results of the current systematic review revealed 2 different imaging examinations could be used to identify or exclude cases with suspected bone metastasis, but the (SEN) and SPE (pooled sensitivity being 0.97 and 0.86, respectively, while pooled specificity was 1.00 and 0.87, respectively) of ^68^Ga-PSMA-PET/CT in the diagnosis of bone metastasis were superior to that of bone scan. ^68^Ga-PSMA-PET/CT has better diagnostic value and more stable results than bone scan because its +LR is far greater than 1 and its -LR is far less than 1. The results of systematic analysis shows that ^68^Ga-PSMA-PET/CT is superior to bone scan in DOR, AUC and *Q∗* index, which indicate that ^68^Ga-PSMA-PET/CT has better diagnostic efficacy than bone scan. However, some studies also found the “Pitfalls” of ^68^Ga-PSMA-PET/CT in the diagnosis of bone metastasis. In a single case study, ^68^Ga-PSMA was absorbed in the skeletal region of Paget disease patients who had been diagnosed by biopsy.^[27]^ This case illustrates recent findings that PSMA may also be overexpressed in tissues other than the prostate cancer, such as myeloma and renal cancer.^[28,29]^ Because the “Pitfalls” of ^68^Ga-PSMA-PET/CT in the diagnosis of bone metastasis of prostate cancer can not be avoided, it is necessary to integrate the PET/CT, bone scan, CT, MRI, PSA, clinical and imaging follow-up, and apply pathology for diagnosis if necessary.

There are some limitations in this study, such as less literature, which reduces the demonstration intensity of meta-analysis; the quality of each study is inconsistent, and the diagnostic threshold is different, which may affect the heterogeneity of this study, and it is also related to the type of included studies. Retrospective studies will reduce the probability of false negative, while prospective studies will increase the probability of false negative and reduce the SPE, but the impact is small. So, this study still needs multi-center, large sample, prospective research to enhance the demonstration intensity.

In conclusion, the ^68^Ga-PSMA-PET/CT has a higher SEN and SPE than bone scan, and it has a higher diagnostic efficiency for prostate cancer bone metastasis. It is worth clinical application. However, we should pay attention to the comprehensive evaluation of patients’ PET/CT, bone scan, CT, MRI, PSA, clinical and imaging follow-up and pathology to avoid the “Pitfalls” of ^68^Ga-PSMA-PET/CT in bone imaging.

## Author contributions

**Conceptualization:** Ruining Zhao, Yajie Li.

**Data curation:** Yajie Li, Lihong Nie.

**Formal analysis:** Yajie Li, Lihong Nie, Hang Zhang, Hongbin Shi.

**Funding acquisition:** Ruining Zhao, Lihong Nie.

**Investigation:** Kaiyue Qin, Hongbin Shi.

**Project administration:** Ruining Zhao.

**Software:** Yajie Li, Lihong Nie, Kaiyue Qin, Hang Zhang.

**Writing – original draft:** Ruining Zhao, Yajie Li, Lihong Nie.

**Writing – review & editing:** Ruining Zhao.
